# Tinnitus: an underreported condition following microvascular decompression for hemifacial spasm

**DOI:** 10.1007/s00701-024-06103-0

**Published:** 2024-05-09

**Authors:** Lina B. M. Albakri, Lilian M. Mennink, Katalin Tamasi, Gea Drost, Pim van Dijk, J. Marc C. van Dijk

**Affiliations:** 1 Department of Neurosurgery, University Medical Center Groningen, PO BOX 30001, 9700RB Groningen, The Netherlands; 2 Department of Epidemiology, University Medical Center Groningen, Groningen, The Netherlands; 3 Department of Neurology and Clinical Neurophysiology, University Medical Center Groningen, Groningen, The Netherlands; 4 Department of Otorhinolaryngology and Head and Neck Surgery, University Medical Center Groningen, Groningen, The Netherlands

**Keywords:** Tinnitus, Microvascular Decompression, Hemifacial Spasm, Quality of Life

## Abstract

**Purpose:**

While hearing loss is a well-known condition following microvascular decompression (MVD) for hemifacial spasm (HFS), tinnitus is an underreported one. This study aims to identify prevalence, characteristics, severity, and predictors of tinnitus following MVD for HFS.

**Methods:**

A single-center cohort of 55 HFS patients completed a questionnaire approximately 5 years following MVD. Data encompassed tinnitus presence, side, type, onset, and severity measured by a 10-point Visual Analogue Scale (VAS). Descriptive, correlation, and logistic regression analyses were conducted.

**Results:**

At surgery, participants’ median age was 58 years (IQR 52–65). The median duration of HFS symptoms before surgery was 5 years (IQR 3–8), slightly predominant on the left (60%). Postoperative tinnitus was reported by 20 patients (36%), versus nine (16%) that reported preoperative tinnitus. Postoperative tinnitus was ipsilateral on the surgical side in 13 patients (65%), bilateral in six (30%), and contralateral in one (5%). Among patients with bilateral postoperative tinnitus, 33% did not have this preoperatively. Tinnitus was continuous in 70% of cases and pulsatile in 30%. Onset of new tinnitus was in 58% immediately or within days, in 25% within three months, and in 17% between three months and one year after surgery. The mean severity of postoperative tinnitus was 5.1 points on the VAS. Preoperative tinnitus and presence of arachnoid adhesions had suggestive associations with postoperative tinnitus in initial analyses (*p* = 0.005 and *p* = 0.065). However, preoperative tinnitus was the only significant predictor of postoperative tinnitus (*p* = 0.011).

**Conclusion:**

Tinnitus is a common condition following MVD for HFS, with a moderate overall severity. Causes behind postoperative tinnitus remain obscure but could be related to those of postoperative hearing loss in this patient population. Clinicians should be aware of tinnitus following MVD and vigilantly monitor its occurrence, to facilitate prevention efforts and optimize outcome for HFS patients undergoing MVD.

## Introduction

Hemifacial spasm (HFS) is characterized by unilateral involuntary facial contractions, involving muscles innervated by the facial nerve (CNVII) [18, 19]. Although not life threatening, HFS substantially impacts quality of life (QoL), often leading to social isolation, depression and imposing high socioeconomic burden [6].

The primary cause of HFS is compression of CNVII at its root exit zone (REZ) at brainstem level in the cerebellopontine angle (CPA). This compression is frequently caused by a vascular loop. The vertebral artery (VA), the anterior inferior cerebellar artery (AICA), and the posterior inferior cerebellar artery (PICA) are most often involved [18, 19]. Due to its anatomical proximity to CNVII, HFS patients may experience symptoms of vestibulocochlear nerve (CNVIII) involvement. These symptoms include vertigo, hearing changes, and tinnitus [31].

The surgical treatment for HFS is microvascular decompression (MVD) of CNVII, a.k.a. the Jannetta procedure [12, 19]. The surgery involves interposing a sponge (Teflon™ or Ivalon®) between the offending vessel and the REZ of CNVII (interposition technique) or repositioning the offending vessel away from the REZ of CNVII (transposition technique) to alleviate compression [12]. Since CNVIII can be inadvertently manipulated during surgery, postoperative hearing changes or even hearing loss (HL) might occur [14].

A lesser-known condition following MVD surgery may be postoperative tinnitus [11, 30]. Like HFS, tinnitus has a negative impact on QoL, often leading to psychosocial problems and loss of productivity [8]. Comprehensive knowledge on the occurrence of tinnitus following MVD surgery for HFS is lacking. In this study, we aim to address this knowledge gap by investigating the prevalence of postoperative tinnitus in HFS patients following MVD. We also aim to identify tinnitus side, type, onset, severity based on impact on QoL, and possible predictors of its occurrence.

## Methods

### Study design and participants

A single-center cohort of 55 HFS patients that underwent MVD between 2015 and 2022 was invited to complete a questionnaire about postoperative tinnitus. Participants were identified based on medical records at an academic tertiary referral center, where the MVD surgery was performed.

### Data collection

Data was collected using a customized questionnaire and by retrieval of relevant information from medical records. The questionnaire was designed to systematically assess postoperative tinnitus. Participants were asked about tinnitus presence, side, type, and onset. They were also asked to rate tinnitus severity based on impact on QoL using a 10-point Visual Analogue Scale (VAS). The VAS ranged from 0 to 10, with 0 representing no perceived impact of tinnitus on QoL and 10 indicating the highest level of perceived impact. Typically, scores between 1 to 3 were considered mild, 4 to 6 moderate, and 7 to 10 severe. Data collected from medical records included demographics, HFS symptoms duration before surgery, side of HFS (i.e., surgical side), time since surgery, presence of arachnoid adhesions, offending vessel(s), decompression technique, and total surgery duration.

### Surgery

All 55 patients were operated by a single surgeon. Surgery consisted of a retrosigmoid craniotomy and the patient’s head was fixed in a Mayfield clamp. After opening the dura, cerebrospinal fluid (CSF) was suctioned from the cisterna magna. The cerebellum was allowed to naturally fall away from the operative field, so no retractors were used. This was followed by cautious dissection of the arachnoid. In cases where arachnoid adhesions were identified intraoperatively, a slightly more aggressive dissection technique was employed to carefully separate the adhered arachnoid layers. The proximal part of CNVII and its REZ were approached caudally, and the compression site was identified with intraoperative neuromonitoring. The compression was then resolved by either the interposition or the transposition technique. The interposition technique was mostly used when a tortuous VA (and occasionally PICA) was the offending vessel. Slight retraction of the artery from the REZ was followed by placement of Teflon™ to isolate the REZ. The transposition technique involved arachnoid dissection of smaller vessels after which the new transposed situation was secured with a Teflon™ patch.

### Data analysis

Data analysis was conducted using SPSS version 29.0 (SPSS, Chicago, USA). Descriptive statistics were used to summarize characteristics of the study population, including medians, interquartile ranges (IQR), counts, and percentages.

Prevalence of pre- and postoperative tinnitus was determined using counts and percentages. To compare the prevalence of tinnitus pre- and postoperatively, a paired non-parametric test (McNemar’s test) was employed. MVD-related tinnitus development/changes were determined on account of cases with newly acquired tinnitus postoperatively, postoperative worsening of preexisting tinnitus, and postoperative improvement of preexisting tinnitus. The association between the side of postoperative tinnitus and the surgical side was examined using Pearson’s chi-square test. The occurrence of different types of tinnitus and the onset of new tinnitus postoperatively were determined using counts and percentages. The mean, median range, and IQR were calculated for the 10-point VAS representing severity of postoperative tinnitus based on impact on QoL. Finally, the occurrence of pre- and postoperative tinnitus in patients in whom arachnoid adhesions were found was specifically determined to assess the influence of these adhesions on tinnitus manifestation and the potential impact of surgical intervention on tinnitus outcomes.

To assess predictors of postoperative tinnitus, binary logistic regression was employed with postoperative tinnitus as dependent variable. Predictors included sex, current age (i.e., age at questionnaire completion), age at surgery, HFS symptoms duration before surgery, side of HFS (i.e., surgical side), time since surgery, presence of arachnoid adhesions, offending vessel(s), decompression technique, total surgery duration, and preoperative tinnitus. Correlations among variables were assessed with Pearson’s correlation coefficients to address multicollinearity. Variables were initially selected based on univariable correlation with the dependent variable using a threshold of p < 0.20. Subsequently, a logistic regression model was constructed. The level of significance for variable inclusion in the final model was set at p < 0.05 based on the results of the multivariable analysis.

## Results

### Demographic and clinical charachteristics

Characteristics of the 55 patients are outlined in Table 1. Males and females were equally divided. At time of MVD surgery, the median age was 58 years (IQR 52–65). The median duration of HFS symptoms before surgery was 5 years (IQR 3–8), with 60% occurring on the left side. The median time since surgery was 5 years (IQR 4–7).

**Table 1:** Demographic and clinical characteristics of the study population

Characteristic	N = 55^*^
Sex	
Female	28 (51%)
Male	27 (49%)
Current Age (in years)	62 (55 – 72)
Age at MVD Surgery (in years)	58 (52 – 65)
HFS Symptoms Duration (in years)	5 (3 – 8)
Side of HFS	
Left	33 (60%)
Right	22 (40%)
Time Since MVD surgery (in years)	5 (4 – 7)
Arachnoid Adhesions	5 (9%)
Offending Vessel(s)	
PICA	21 (38%)
AICA	8 (15%)
VA	3 (6%)
PICA and AICA	6 (11%)
PICA and VA	12 (22%)
AICA and VA	5 (9%)
Decompression Technique	
Interposition	39 (71%)
Transposition	16 (29%)
Total Surgery Duration (in minutes)	131 (107 – 161)

^*^Median (IQR); n (%)

MVD: Microvascular Decompression, HFS: Hemifacial Spasm, PICA: Posterior Inferior Cerebellar Artery, AICA: Anterior Inferior Cerebellar Artery, VA: Vertebral Artery

Arachnoid adhesions were found in 9% of patients. The offending vessel was PICA in 38%, AICA in 15%, VA in 6%, both PICA and VA in 22%, both PICA and AICA in 11%, and both AICA and VA in 9%. The interposition technique was employed in 71% of cases, versus 29% where the transposition technique was used. The median total surgery duration was 131 min (IQR 107–161).

### Tinnitus

**Fig. 1:**
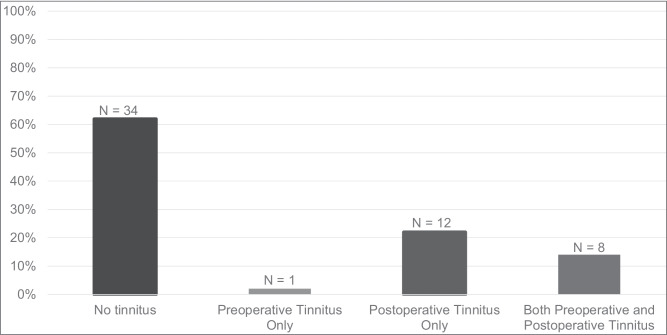
Prevalence of tinnitus among patients that underwent MVD for HFS, including counts and percentages of patients with: no tinnitus, preoperative tinnitus only, postoperative tinnitus only, and both preoperative and postoperative tinnitus (total: n = 55)

The prevalence of tinnitus pre- and postoperatively is displayed in Fig. 1. Preoperative tinnitus was reported by nine patients (16%), whereas 20 patients (36%) reported postoperative tinnitus. One patient that had preoperative tinnitus, reported postoperative recovery. McNemar’s test revealed a statistically significant difference between the prevalence of tinnitus pre- and postoperatively (*p* = 0.003).

**Fig. 2:**
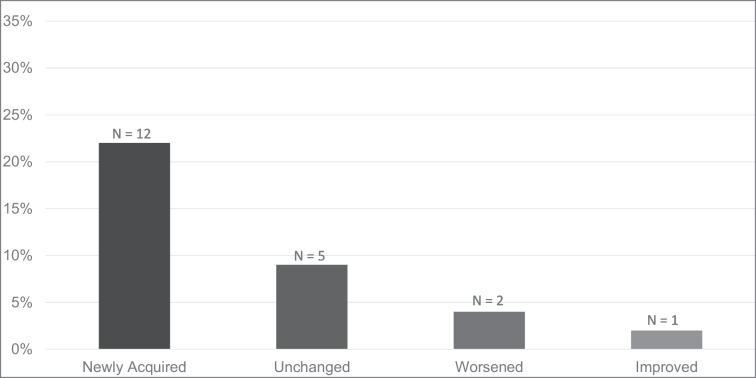
Postoperative tinnitus subcategories among patients that underwent MVD for HFS, including newly acquired tinnitus, preoperative tinnitus that remained unchanged, preoperative tinnitus that worsened, and preoperative tinnitus that improved postoperatively. The total count and percentage is of patients with postoperative tinnitus (n = 20/55; 36%)

Figure 2 shows postoperative tinnitus subcategories that included: 1) newly acquired tinnitus postoperatively (n = 12; 22%); 2) preoperative tinnitus that remained unchanged postoperatively (n = 5; 9%); 3) preoperative tinnitus that worsened postoperatively (n = 2; 4%); and 4) preoperative tinnitus that improved postoperatively (n = 1; 2%). Table 2 presents pre- and postoperative tinnitus status comparison.

**Table 2:** Pre- and postoperative tinnitus status comparison

Preoperative tinnitus	N	Postoperative tinnitus	N
Yes	9	No	1
		Yes, unchanged	5
		Yes, worsened	2
		Yes, improved	1
No	46	No	34
		Yes, newly acquired	12
Total	55		55

Thirty-four patients (62%) never experienced tinnitus, neither before nor after the surgery (Table 2/Fig. 1). Forty patients (73%) did not experience MVD-related tinnitus development or changes, which was on account of the absence of newly acquired tinnitus postoperatively or postoperative worsening or improvement of preexisting tinnitus. The remaining 15 patients (27%) had MVD-related tinnitus development or changes, encompassing new tinnitus postoperatively (22%), postoperative worsening of preexisting tinnitus (4%), and postoperative improvement of preexisting tinnitus (2%) (Table 2/Fig. 2**)**.

Of all 20 patients with postoperative tinnitus, 13 patients (65%) had it ipsilaterally on the surgical side, six patients (30%) had it bilaterally, and one patient (5%) had it on the contralateral side. Of the six patients with bilateral tinnitus, four (67%) had that already preoperatively and two (33%) developed it bilaterally only after the surgery. There was a statistically significant association between the side of postoperative tinnitus and the surgical side (*χ*^*2*^ = 10.976, *df* = 2, *p* = 0.004). Fourteen patients (70%) reported continuous tinnitus, and six patients (30%) reported pulsatile tinnitus. One patient with continuous tinnitus reported gaze-modulated tinnitus, in which the tone frequency of tinnitus varied with eye movements.

**Fig. 3:**
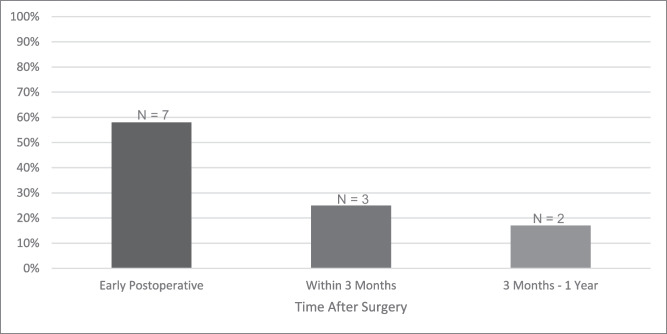
Onset of newly acquired postoperative tinnitus among patients that underwent MVD for HFS, categorized into three time intervals: 1) immediately or within the first few days after surgery (early postoperative); 2) within three months after surgery; and 3) between 3 months and one year after surgery. The total count and percentage only include patients with newly acquired tinnitus postoperatively (n = 12)

Figure 3 presents the onset of newly acquired tinnitus postoperatively. Of the 12 patients with new postoperative tinnitus, seven (58%) experienced it directly or within the first few days; three (25%) experienced it within the first three months; and two (17%) experienced it between three months and one year after surgery.

The 10-point VAS representing postoperative tinnitus severity based on impact on QoL ranged widely among participants from a minimum of 1.0 point to a maximum of 9.8 points (IQR 2.5–7.4). The median severity score was 5.0 points, which closely aligns with the mean of 5.1 points (i.e., moderate severity).

### Tinnitus in relation to arachnoid adhesions

Out of all 55 patients, arachnoid adhesions were found in five patients during surgery. Of those, three patients had tinnitus preoperatively and four reported having it postoperatively. Two of the patients with postoperative tinnitus had it ipsilaterally on the surgical side, and two had it bilaterally. In one of the patients that had tinnitus ipsilaterally, the tinnitus was newly acquired postoperatively. The other patient with ipsilateral tinnitus, along with one of the two patients with bilateral tinnitus, experienced postoperative worsening of preexisting tinnitus. The other patient with bilateral tinnitus experienced no postoperative change in preexisting tinnitus.

### Predictors of postoperative tinnitus

Table 3 shows the results of regression analyses aimed at identifying predictors of postoperative tinnitus. Addressing multicollinearity, several variables were highly correlated. Not surprisingly, current age and age at MVD surgery had a strong positive correlation (*Pearson's r* = 0.968, *p* < 0.001). Additionally, HFS symptom duration and surgery duration had a significant positive correlation (*Pearson's r* = 0.401, *p* = 0.003). Moreover, side of HFS and arachnoid adhesions had a significant positive correlation (*Pearson's r* = 0.387, *p* = 0.003), as individuals with HFS on the right side were more likely to have arachnoid adhesions. Side of HFS also had a significant positive correlation with preoperative tinnitus (*Pearson's r* = 0.341, *p* = 0.011), as patients with HFS on the right side were more likely to have preoperative tinnitus. As such, arachnoid adhesions and preoperative tinnitus had a significant positive correlation (*Pearson's r* = 0.373, *p* = 0.005). Other significant correlations included: side of HFS and offending vessel (*Pearson's r* = –0.299, *p* = 0.027), side of HFS and decompression technique (*Pearson's r* = –0.360, *p* = 0.007), offending vessels and decompression technique (*Pearson's r* = 0.375, *p* = 0.005), decompression technique and surgery duration (*Pearson's r* = 0.375, *p* = 0.005), and decompression technique and preoperative tinnitus (*Pearson's r* = –0.283, *p* = 0.036).

**Table 3:** Uni- and multivariable logistic regression analyses of predictors of postoperative tinnitus

Predictor Variable	No Postoperative Tinnitus N = 35^*^	Postoperative Tinnitus N = 20^*^	Univariable Analyses		Multivariable analysis	
			Coefficient (95% CI)	p-value	Coefficient (95% CI)	p-value
Sex			0.373 (− 0.727—1.473)	0.508	-	-
Female	19 (54%)	9 (45%)				
Male	16 (46%)	11 (55%)				
Current Age (in years)	62 (54 – 71)	67 (61 – 73)	0.037 (− 0.026—0.100)	0.239	-	-
Age at MVD Surgery (in years)	58 (48 – 65)	62 (53 – 67)	0.038 (− 0.025—0.101)	0.226	-	-
HFS Symptoms Duration (in years)	5 (4 – 8)	5 (2 – 10)	− 0.002 (− 0.098—0.094)	0.976	-	-
Side of HFS			0.325 (− 0.791—1.441)	0.568	-	-
Left	22 (63%)	11 (55%)				
Right	13 (37%)	9 (45%)				
Time Since MVD surgery (in years)	6 (3 – 7)	5 (4 – 7)	− 0.017 (− 0.272—0.238)	0.896	-	-
Arachnoid Adhesions	1 (3%)	4 (20%)	**2.140 (− 0.131—4.411)**	**0.065**	1.366 (− 1.236—3.602)	0.304
Offending Vessel(s)			0.109 (− 0.187—0.405)	0.469	-	-
PICA	15 (43%)	6 (30%)				
AICA	4 (11%)	4 (20%)				
VA	2 (6%)	1 (5%)				
PICA and AICA	4 (11%)	2 (10%)				
PICA and VA	8 (23%)	4 (20%)				
AICA and VA	2 (6%)	3 (15%)				
Decompression Technique			− 0.318 (− 1.55—0.920)	0.614	-	-
Interposition	24 (69%)	15 (75%)				
Transposition	11 (31%)	5 (25%)				
Total Surgery Duration (in minutes)	132 (103 – 161)	130 (109 – 168)	0.004 (− 0.016—0.024)	0.568	-	-
Preoperative Tinnitus	1 (3%)	8 (40%)	**3.121 (0.940—5.302)**	**0.005**	**2.882 (0.664—5.100)**	**0.011**

^*^Median (IQR); n (%)

CI: Confidence Interval, MVD: Microvascular Decompression, HFS: Hemifacial Spasm, PICA: Posterior Inferior Cerebellar Artery, AICA: Anterior Inferior Cerebellar Artery, VA: Vertebral Artery

Based on univariable logistic regression analyses, only arachnoid adhesions and preoperative tinnitus had a suggestive association with postoperative tinnitus (*p* < 0.20) and were thus included in the multivariable analysis. According to the multivariable analysis, preoperative tinnitus was the sole significant predictor of postoperative tinnitus (*coefficient* = 2.882, *p* = 0.011).

## Discussion

This study aimed to investigate the prevalence of postoperative tinnitus in HFS patients that underwent MVD, its characteristics, severity based on impact on QoL, and predictors of its occurrence. It was found that a significant proportion of HFS patients experienced pre- and postoperative tinnitus. This suggests that a shared underlying mechanism or vulnerability of both CNVII and CNVIII might be present in HFS patients. Preoperatively, 16% of patients experienced tinnitus. This compares to the prevalence in the general population in the same age group, which is around 13% [29]. Postoperatively, tinnitus was reported by 36% of patients, which is considerably higher than the prevalence in the general population in the same age group.

### Preoperative tinnitus

Although the prevalence of preoperative tinnitus in our cohort is comparable to the general population, there is evidence that tinnitus and other vestibulocochlear symptoms may be associated with HFS due the presence of a neurovascular conflict. A study by Di Stadio et al. reported the correlation between characteristics of vascular loops in the CPA and symptoms of HFS, HL, tinnitus, and vertigo. It was found that vascular loops with a diameter > 0.85mm that are in direct contact with CNVIII are significantly associated with vertigo, tinnitus, and HFS. Sensorineural HL correlated with the number and lengths of contacts between the vascular loop and CNVIII [31]. This suggests that, depending on its characteristics, a vascular loop compressing CNVII and resulting in HFS may also influence CNVIII, leading to vertigo, tinnitus, and/or HL in HFS patients.

Microvascular compression of CNVIII has been proposed as a pathophysiological mechanism for tinnitus, with MVD of CNVIII potentially serving as a curative treatment [20, 26]. One study showed that patients with both HFS and tinnitus had a neurovascular conflict of both CNVII and CNVIII, while this was less present in HFS patients without tinnitus. Tinnitus resolved or markedly improved after MVD of CNVIII in many cases [28]. Nevertheless, MVD of CNVIII for tinnitus was shown not to be beneficial in a meta-analysis by van den Berge et al. [2].

In our cohort, arachnoid adhesions were observed in some cases during surgery. Arachnoid adhesions also appeared to have a statistically significant positive correlation with preoperative tinnitus. Notably, inflammation is a pathophysiological mechanism in which a membrane becomes adhesive to itself and surrounding structures. Arachnoid adhesions are thereby suggestive of inflammation, which is the primary cause for abnormal appearances of the arachnoid [7]. Thickened, opaque, and sticky arachnoid membranes have sometimes been observed during MVD surgery for HFS. One study found that blood inflammatory markers were elevated in HFS patients compared to controls, suggesting that inflammation might play a role in HFS [5]. Inflammation has also been suggested to play a role in tinnitus. Inflammatory markers such as TNF-α and IL-1β were found to be increased in tinnitus, and inflammatory cells such as microglia and astrocytes were found to be activated. Inflammation may lead to alterations in synaptic transmission and synaptic organization, leading to tinnitus, which has been associated with an increase in excitatory and a decrease in inhibitory neurotransmission [15]. Thereby, inflammation is another conceivable mechanism for preoperative tinnitus in HFS patients. Also, arachnoid adhesions may cause mechanical compression of CNVII and CNVIII. In line with this concept, some studies have stated that only complete separation and dissection of arachnoid membranes can result in sufficient decompression of CNVII for HFS [5, 23].

### Postoperative tinnitus

Several events during MVD surgery may lead to CNVIII injury, potentially resulting in postoperative HL, tinnitus and/or vertigo. This includes ischemic, mechanical, dehydrating, or thermal trauma to the nerve [10]. While HL often is the result of injury to the proximal part of CNVIII and higher auditory pathways, it has been suggested that tinnitus is a result of injury to the cochlear (distal) part of the nerve [11]. Other potential mechanisms for postoperative tinnitus include pneumocephalus, which can result in pulsatile tinnitus due to communication between the drilled mastoid and the epidural space created during surgery [9].

A high prevalence of tinnitus following CPA tumor surgery has been reported [3, 17]. Such tinnitus might be related to the (para)flocculus [17]. The flocculus and paraflocculus are small lobes of the cerebellum, responsible for gaze maintenance during head movement [16]. It has been found that flocculus volume is decreased in patients after CPA tumor removal, due to compression of the tumor or manipulation of the flocculus during surgery. Related postoperative tinnitus is often gaze-modulated, in which patients can change loudness or pitch of their tinnitus by ocular movements [17]. Surgical manipulation of the cerebellum may also take place during MVD surgery for better visualization [12, 19, 30]. Although in our study population cerebellar retraction was avoided, manipulation of the flocculus during the procedure cannot be excluded. This might explain why one patient in our cohort reported gaze-modulated tinnitus postoperatively.

Although postoperative tinnitus was mainly reported on the surgical side, some patients reported bilateral tinnitus. It is conceivable that contralateral tinnitus has a similar mechanism as that for contralateral HL following posterior cranial fossa surgery. This indicates that other causes than direct surgical manipulation are involved. Mechanisms for contralateral HL include drill noise, brain shift, vascular compromise, autoimmunity, venous infarction, saline overdose, meningitis, general anesthesia with nitric oxide, and ototoxic drugs [32]. Endolymphatic hydrops affecting the inner ear due to CSF loss during surgery, may also contribute to bilateral HL [32, 33]. It is thereby possible that comparable mechanisms contribute to contralateral postoperative tinnitus in this patient population. This, however, needs to be further investigated.

Most patients experienced newly acquired postoperative tinnitus immediately or within the first few days after surgery, indicating a plausible association between the surgery and the development of tinnitus. Other patients however, reported the onset of tinnitus several months later. This delayed tinnitus could possibly be compared to delayed HL after MVD surgery, which has been defined as occurring more than 45 days postoperatively [13]. Although delayed HL has occasionally been reported, its rarity makes it difficult to identify the exact cause. The same could apply to delayed tinnitus. In some studies, delayed HL following MVD was treated with corticosteroids and patients showed improvement after two months [13, 21]. This, again, may point towards inflammation, as an inflammatory reaction could take place in the operated area postoperatively. Such reaction following an MVD procedure could affect the function of both CNVII and CNVIII [25]. In addition, there have been many reports of delayed facial palsy after MVD for HFS. One study did illustrate that delayed HL and facial palsy may occur simultaneously, suggesting that these two conditions may have a similar etiology [13]. Moreover, it is established that the pathophysiology of tinnitus is closely related to that of HL [15], meaning that all three conditions may share a common pathophysiological mechanism. However, there is still a lack of clear evidence in this regard.

The VAS for tinnitus severity based on impact on QoL ranged widely among patients with a mean of 5.1 points, indicating moderate severity. However, it is important to note that the perception of tinnitus severity and impact on QoL is highly individual, explaining the wide range among patients. Factors such as coping mechanisms, lifestyle, psychological state, and general health can influence how affected patients experience their tinnitus [4]. Therefore, while tinnitus appears to be a prevalent condition among HFS patients that underwent MVD, the perception of its severity spans the whole VAS and is highly patient-dependent.

### Predictors of postoperative tinnitus

Multivariable regression analysis revealed preoperative tinnitus as the sole significant predictor of postoperative tinnitus. Nevertheless, this analysis is contingent upon a limited patient sample size, potentially leading to the absence of other significant predictors. Our findings do, however, suggest that HFS patients that present with tinnitus preoperatively are most likely going to keep on experiencing it postoperatively. This emphasizes the importance of preoperative assessment and counseling to manage expectations. Moreover, despite the proven age-dependent occurrence of HL, no such dependency was proven for tinnitus [22], which was also evident in our regression analyses. Like age-related HL, tinnitus has been shown to be more common in males than females [1, 27]. This, however, was not evident in our analyses, most likely due to the small sample size.

### Limitations and future perspectives

This study has several limitations. First, it is a retrospective cohort study with a relatively small sample size from a single center, which may limit the generalizability of the findings. Second, although the pathophysiology of tinnitus is closely related to that of HL, we did not take that into account. Incorporating pre- and postoperative data on hearing function would be valuable in future investigations. Third, intraoperative brainstem auditory evoked potentials (BAEP) monitoring, which is used to monitor CNVIII and higher auditory pathways to prevent postoperative HL, could also be used to predict and prevent postoperative tinnitus. We however did not conduct detailed analyses of intraoperative BAEP peaks and their correlation to postoperative tinnitus. This is because in the period of 2015–2022, BAEP monitoring was still in a developing stage in our center and most intraoperative BAEP data could not be used to draw accurate conclusions. Incorporating intraoperative BAEP data and their correlation to postoperative tinnitus would thus be of great value in future investigations. Fourth, data on tinnitus prevalence, onset and characteristics were collected through questionnaires approximately 5 years post-MVD. This introduces the possibility of recall bias, as participants may have difficulty accurately recalling such events over an extended period. Prospectively collecting data on tinnitus pre- and post-MVD would be a better approach in future investigations. Finally, although VAS scales have been shown to be a valid and reliable screening tool for obtaining information about tinnitus severity, validated tinnitus questionnaires such as the tinnitus functional index (TFI) or the tinnitus handicap inventory (THI) are considered more reliable for identifying tinnitus severity [24]. The TFI or the THI should thus be filled in by HFS patients pre- and post-MVD to get a more comprehensive understanding of the impact of tinnitus on QoL in this patient population. Therefore, future research should involve larger prospective cohorts, incorporating pre- and postoperative hearing tests, intraoperative BAEP findings, and validated tinnitus questionnaires data to validate our findings and to explore potential interventions to mitigate tinnitus in HFS patients undergoing MVD surgery.

## Conclusion

In our cohort, tinnitus was revealed as a common condition following MVD surgery for HFS. Its impact on QoL varies widely among patients, with a moderate overall impact. The etiology of postoperative tinnitus is still unknown but could be closely related to postoperative HL. CNVIII injury during surgery, pneumocephalus, flocculus manipulation, endolymphatic hydrops, and inflammation are plausible pathophysiological mechanisms. Long-term follow-up with monitoring of auditory function in operated HFS patients is essential to identify the course and timing of tinnitus development, to provide insights into its etiology and pathophysiology. Further research is warranted to refine risk prediction for postoperative tinnitus.

## Data Availability

The dataset generated and analyzed during the current study is not publicly available due to sensitivity of the data and restrictions from the informed consent, but is available from the corresponding author upon reasonable request.

## Abbreviations

HFS: Hemifacial Spasm
CNVII: Facial Nerve
QoL: Quality of Life
REZ: Root Exit zone
CPA: Cerebellopontine Angle
VA: Vertebral Artery
AICA: Anterior Inferior Cerebellar Artery
PICA: Posterior Inferior Cerebellar Artery
CNVIII: Vestibulocochlear Nerve
MVD: Microvascular Decompression
HL: Hearing Loss
VAS: Visual Analogue Scale
CSF: Cerebrospinal Fluid
IQR: Interquartile Range
CI: Confidence Interval
TFI: Tinnitus Functional Index
THI: Tinnitus Handicap Inventory
